# The Best Means of Preserving the Eye-Sight of Dental Practitioners

**Published:** 1873-01

**Authors:** 


### The Best Means of Preserving the Eye-Sight of Dental Practitioners.

*Read Before the Ohio State Dental Society.*

This is indeed an important subject, and especially important to the dentist, involving as it does his own happiness and professional usefulness, and the comfort of his family.

It is important alike to the young and to the middle aged. The former class are anxious to preserve their sight, and the latter class are equally anxious to postpone the characteristics of old age, at least as far as their eyesight is concerned. Permit me to give a brief description of the eye as given by Prof. A. Metz, in his anatomy and histology of the eye. The human eye is an organ-physical apparatus which has, by means of a system of collective media, the property of casting real images of objects on the retina. The impression of light thus made on the nerve-membrane is conducted by the optic nerve-fibres to the brain, where consciousness is enforced. This wonderful little dioptric organ ranges, anatomically, over a wide field, containing within itself and its appendages all the structures composing the human body. As an optical instrument it is perfect beyond imitation, having the wonderful property of self adaptation to long or short distances.

In order to apply a remedy in any diseased condition, it is always necessary to know what is the cause of the disease, and why it is produced. The work of the dentist is such as to require the adjustment of the eye to a certain position, and also demands that this position be maintained for a considerable length of time. This constant looking at small objects near the eye demands a convergence of the sight, and this convergence is produced by the contraction of the internal recti muscles. You all know that by constantly using a muscle we are liable to strain and overwork it, producing weakness and want of tone. This being true, the work of the dentist has a tendency to produce muscular asthenopia, and acaommodative asthenopia. By asthenopia we mean the inability of maintaining the adjustment of the eye for short distances for a length of time, and the hyperæsthesia of the retina and ciliary nerves. The dentist is liable to have muscular asthenopia on account of constantly using the internal recti muscles while operating, being compelled to contract these muscles in order to see a small object near the eye, and the same position of the eye is maintained in excavating a cavity or in filling the same. He is also liable to have accommodative asthenopia by constantly looking at a small object near the eye, as you know we do not materially change the distance from the eye to the object during an operation. These two forms of the same disease should be avoided and dreaded by the dentist, because his daily work has a direct tendency to produce one or both. If you have muscular asthenopia you can detect it by the eye becoming weary while looking at a small object for a length of time, and also by one or both eyes refusing to maintain a converged position. You will know by this test that one or both of the internal recti muscles are either sprained or overworked, and that they are not sufficiently strong to maintain this converged position of the eye necessary in looking at a small object near the eye, or in operating. If you have accommodative asthenopia, you may detect it by the eyes becoming weary while reading, and the letters will seem blurred and indistinct.

I have consulted a number of authors as to the cause of asthenopia, and I find that they all agree that the two forms of this disease are produced by overworking the muscles of accommodation or the internal recti muscles. This accommodative act is exceedingly interesting and instructive. This accommodation of the eye to different focal distances is controlled and produced by the action of the ciliary muscle; that is, when we wish to see an object at a certain distance from the eye this muscle acts upon the lens in such a manner as to enable us to see the object distinctly. The accommodative act is nicely illustrated in looking at stereoscopic views. When you first place the stereoscope before the eye the view is not distinct as it is after you hold the instrument in position for a few seconds. I trust you will, by this illustration, comprehend what is meant by the muscular accommodation of the eye which enables us to see objects distinctly at different focal distances, and also that you may see how this muscle of accommodation may become overworked, weakened, and diseased by constantly looking at an object at a certain distance from the eye, or by doing just such work as the dentist is daily called upon to do. I can perhaps more perfectly illustrate how this muscle of accommodation may become diseased by giving you the position it maintains, and the function it is required to perform in making a dental operation.

In looking at a small object near the eye we have contraction of the circular or voluntary fibres of the ciliary muscle, causing an increased anteroposterior diameter of the lens, that is the anterior convexity of the lens is increased. This muscular contraction must of course be maintained during the entire operation. The important question with the dentist is, how to avoid these two forms of this disease; also how to obtain relief when suffering from its effects.

The various authors on diseases of the eye agree that to prevent this disease we must have strong, steady light; frequent change of position while working; also frequent interruptions so as to rest the eye. When we first detect the presence of this disease, we should immediately use convex glasses. These glasses are especially valuable in relieving accommodative asthenopia. By their use the object is magnified, and this enlarging the object will decrease the labor of the ciliary muscle, or muscle of accommodation. These glases will also relieve the internal recti muscles, but not to the same extent as the muscle of accommodation. The best way to relieve the internal recti muscles is to use convex glasses ground in with prismatic glasses. The use of these glasses will relieve the convergence of the eyes, and of course will relieve the muscle producing the convergence. Another mode of relieving muscular asthenopia is recommended by some authors, called tenotomy. This operation consists in clipping the tendon of the external rectus, so as to enable the internal recti muscles to converge the eyes with less effort. The objection to this operation, is if the tenotomy be too extensive, a convergent squint is likely to be produced.

In order to avoid this disease, as I have suggested, have strong steady light; frequent change of position while operating; and rest the eye often looking around the room or at some distant object. If you have accommodative asthenopia, use convex glasses so as to magnify the object, and in muscular asthenopia use convex glasses ground in with prismatic glasses, and in accommodative asthenopia combined with muscular asthenopia, use convex glasses combined with prismatic glasses. This, of course, is a work for oculists, who are the proper persons to consult.

